# Rotatable precipitates change the scale-free to scale dependent statistics in compressed Ti nano-pillars

**DOI:** 10.1038/s41598-019-40526-5

**Published:** 2019-03-07

**Authors:** Yan Pan, Haijun Wu, Xiaofei Wang, Qiaoyan Sun, Lin Xiao, Xiangdong Ding, Jun Sun, Ekhard K. H. Salje

**Affiliations:** 1 State Key Laboratory for Mechanical Behavior of Materials, Xi’an Jiaotong University, Xi’an, 710049 China; 2 Department of Materials Science and Engineering, National University of Singapore, Singapore, 117575 Singapore; 3 Department of Earth Sciences, University of Cambridge, Cambridge, CB2 3EQ United Kingdom

## Abstract

Compressed nano-pillars crackle from moving dislocations, which reduces plastic stability. Crackling noise is characterized by stress drops or strain bursts, which scale over a large region of sizes leading to power law statistics. Here we report that this “classic” behaviour is not valid in Ti-based nanopillars for a counterintuitive reason: we tailor precipitates inside the nano-pillar, which “regulate” the flux of dislocations. It is not because the nano-pillars become too small to sustain large dislocation movements, the effect is hence independent of size. Our precipitates act as “rotors”: local stress initiates the rotation of inclusions, which reduces the stress amplitudes dramatically. The size distribution of stress drops simultaneously changes from power law to exponential. Rotors act like revolving doors limiting the number of passing dislocations. Hence each collapse becomes weak. We present experimental evidence for Ti-based nano-pillars (diameters between 300 nm and 2 μm) with power law distributions of crackling noise P(*s*) ∼ *s*^−*τ*^ with *τ* ∼ 2 in the defect free or non-rotatable precipitate states. Rotors change the size distribution to P(*s*) ∼ exp(−*s*/*s*_0_). Rotors are inclusions of ω-phase that aligns under stress along slip planes and limit dislocation glide to small distances with high nucleation rates. This opens new ways to make nano-pillars more stable.

## Introduction

Building progressively smaller technological devices requires revaluation of size-independent material engineering^1^. A main problem is brittleness at small scales in metals, alloys, multi-ferroics and other functional materials. Experimental observations first indicated that “smaller is stronger”^2^, but then it was found that intermittent stress singularities (jerks) also increase. Such tendency that “smaller is wilder”^3^ contrasts Gaussian plastic fluctuations in large samples^3^ with scale-free intermittency at micro and nano scales for the same materials^4–10^. The abrupt strain jumps in quasi-statically loaded micro-/nano-components endanger structural stability and the associated unpredictability raises serious challenges for applications^10,11^.

Stress serrations of nano-pillars are common^6,7,12–14^ when stress drops, energies, and waiting times are power law distributed with universal properties^4,15–21^. The underlying atomic mechanism of serration is often the propagation of dislocations along slip planes inside nano-pillars. To avoid this inhomogeneous plastic slip, various inclusions have been introduced to inhibit dislocation avalanche propagation. In duralumin pillars, the slip steps on the 1 μm pillar are smaller and more homogeneously distributed, compared with the Al counterpart^22^, which suggests smaller strain burst behaviour with precipitation hardening in pillars. However, below several micron, intermittent plastic flow is also observed in duralumin micropillars^22^ and Ni-based oxide-dispersion strengthened alloys^23^. In addition, there are also experimental observations indicating that strain bursts are promoted by introducing nanoscale *η*’ phase to Al micropillars^24^, because a number of dislocations are first trapped by precipitates, and then suddenly move in a correlated fashion, leading to large bursts. However, here we report that the number of serrations, their energy distribution and their inter-event times can be tailored in Ti-based nano-pillars by the inclusion of pinning centres that reduce dislocation movements. Our pinning centres are small inclusions of ω-phase, which can easily be introduced by appropriate heat treatment and slight changes of the chemical composition. We observed that rotating ω-phase inclusions change crackling noise from power-law serration to exponential distributions of stress drops and waiting times between stress drops. This is of fundamental importance: we know so far of no process that destroys the space and time invariance of stress drops^25–28^ in such a simple doping process. Stress drop mechanisms and related processes were discussed in some theoretical papers^27–32^ while simple analytical theories always lead to power law singularities^33–35^. We have discovered a material where we can switch between scale invariant behaviour in the power law regime to cut-off dominated processes with well-defined energy and time scales. The wider implication is that we are now able to improve the properties of nano-pillars to make them stronger and more stable^36,37^. We will show that compression in rotor-stabilized nano-pillars does not lead to macroscopic slip band and show smooth strain-stress curves.

## Materials Design

**Figure 1:**
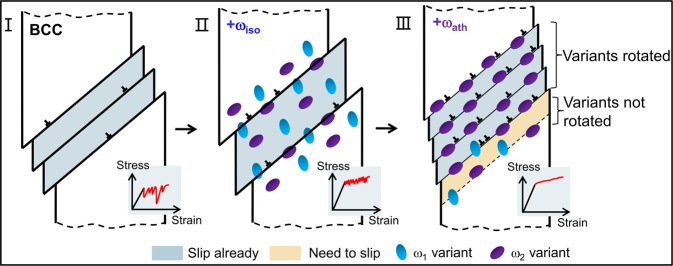
Design concept for avalanches and yield strength engineering in β-Ti alloy nano-pillars. The three scenarios are: no ω phase precipitates and large avalanches (I), inclusions of ω_iso_ precipitates and reduced avalanches (II), and rotor inclusions of ω_ath_ precipitates with even smaller fluctuations (III).

ω inclusions in β-Ti alloys are either a metastable athermal phase, which is produced by rapid quenching, or isothermal phases when samples are aged at appropriate temperatures. Small sized athermal ω (ω_ath_) precipitates form by local collapse of the {111} planes of the β phase (BCC structure) via a shuffle mechanism with four crystallographic variants^38^. The size range of the precipitates is 2–10 nm. The isothermal ω (ω_iso_) precipitates grow from aged ω_ath_ inclusions. The growing ω_iso_ precipitates coarsen and reject stabilizing elements into the surrounding β matrix^39,40^. Thus, the ω_iso_ precipitate is a stable phase, while ω_ath_ precipitate represents a metastable phase with the same composition as the β matrix^39,40^. We make use of the different properties of ω precipitates to influence dislocation movements. Three scenarios were designed to represent different degrees of confinement and dynamics of dislocation movements (Fig. 1). In the first scenario of BCC nano-pillars without precipitates, dislocations can travel freely and stress drops are large. The stress-strain curves (displacement-controlled loading) show large stress fluctuations during deformation^7,41^. In the second scenario, ω_iso_ precipitates hinder the movement of dislocations and, thus, the amplitudes of stress drops are reduced^14,42^. In the third scenario, ω_ath_ precipitates further reduce the drop amplitudes and keep high yield strength. These ω_ath_ precipitates act as rotors and enable nucleation of dislocations so that a very large number of small dislocations with many dense slip lines coexist and the strain-stress curve becomes almost smooth.

Experimentally, nano-pillars of β-Ti alloys (Ti-32Mo, Ti-20Mo and Ti-10V-2Fe-3Al) were manufactured with sizes between 300 nm and 2 μm and bulk compositions: (a) Ti-32Mo without ω phase precipitates, (b) Ti-20Mo with ω_iso_ precipitates, and (c) Ti-10V-2Fe-3Al with ω_ath_ precipitates (ω_ath_-Ti-1023). The detailed heat treatment procedures are described in the section on sample preparation. Microstructures were characterized with transmission electron microscope (TEM) for the three types of β-Ti alloys, as shown in the Supplementary Fig. S1. Both ω_ath_ and ω_iso_ precipitates in the present cases have similar mean diameter of 3∼4 nm and similar mean spacing of 6∼8 nm.

## Results

### Comparison of mechanical behavior

**Figure 2:**
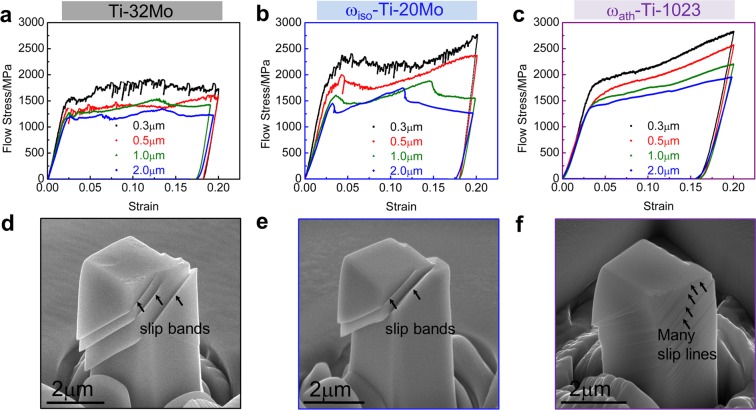
Compression test on β-Ti alloy nano-pillars. (**a**–**c**) Comparison of mechanical behavior for the three scenarios in Fig. 1 and different pillar sizes. (**d**–**f**) The SEM images show the equivalent slip configurations for the three alloys of 2 μm pillars.

Figure 2 shows the effect of precipitates on compression in three types of nano-pillars (Ti-32Mo, ω_iso_-Ti-20Mo, and ω_ath_-Ti-1023 alloy), additional strain-stress curves are shown in Supplementary Fig. S2. The “jerkiness” of the strain-stress curve decreases from (a) to (b) at the large pillar sizes, while the reduction becomes not obvious at small pillar sizes. The “jerkiness” dramatically decreases in (c) at all pillar sizes. It indicates that ω_iso_ can reduce the stress fluctuations at the large pillar sizes while the ω_ath_ can reduce the stress fluctuations at all pillar sizes. It is noted that the stress-strain curves of the ω_iso_-Ti-20Mo alloy (Fig. 2b) show obvious stress oscillation, especially for 1.0 and 2.0 µm pillars, which is caused by the activation of large slip bands^42^. In contrast, the stress-strain curves of ω_ath_-Ti-1023 alloy (Fig. 2c) exhibit continuous stable plastic flow phenomenon. Thus, we simply calculate the slope of the curve at stress increasing region from 6% to 11% to represent the ω strengthening effect. The calculated slopes in ω_iso_-Ti-20Mo alloy are 6040 MPa for 2.0 µm pillar and 5308 MPa for 1.0 µm pillar, while the slopes in ω_ath_-Ti-1023 alloy are 3660 MPa for 2.0 µm pillar and 3886 MPa for1.0 µm pillars. The result shows that ω_iso_ precipitate exhibit a higher strengthening effect and a less stable effect on plasticity than the ω_ath_ precipitate. After deformation, multiple large slip bands were found after deformation in (a), (b), and many slip lines (but no slip bands) with small amplitudes occur on the pillar surface in (c). Only one slip system is activated when the samples are deformed in <011>. The slip planes have angles of ca.52° with the {011} top surface and represents the {112} <111> slip system.

### Statistics for stress drops and waiting times

**Figure 3:**
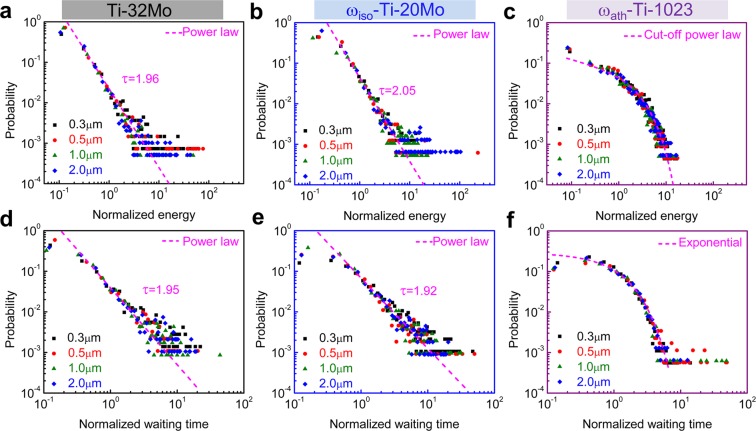
Probability distribution functions for stress drops and waiting times for the three scenarios in Fig. 2. (**a**–**c**) The stress drops are normalized by the average stress drop, which rescales all curves to be independent of the pillar diameter. The fitting line in (**c**) is cut-off power law P(*s*) = *s*^−1.3^exp(−*s*/2.68) ∗ (1 − exp(−*s*/15.64)). (**d**–**f**) The waiting times are normalized by the average waiting time, which rescales all curves to be independent of the pillar diameter. The fitting line in (**f**) is exponential distribution with P(*s*) = 0.29 ∗ exp(−*s*/*s*_*o*_), where *s*_*o*_ is normalized to 1.0.

The stress-strain curves in Fig. 2 were analysed by calculating the squared temporal derivatives of the stress: (d*σ*/d*t*)^2^ ^30,31,43^. The detailed calculations are described in the section of statistics method and Supplementary Fig. S3. The avalanche statistics are shown for the three cases in Fig. 3a–c containing some very large events, which correspond to spanning slips, and a multitude of smaller events of dislocation glide. Only the latter are included in the probability distribution function (PDF) because the sizes of the largest stress drops are determined by boundary conditions (spanning avalanches from surface to surface). The PDFs are strongly size dependent and collapse onto a master curve when the amplitudes are normalized by the average amplitude for each system.

Power law distributions were found only for the first two scenarios. The fitted power law exponents are almost identical: 1.96 and 2.05 with the incorporation of ω_iso_ precipitates. A drastic change of the PDF occurs in the third scenario when ω_ath_ precipitates are introduced. Crackling noise of stress drops (measured as normalized (d*σ*/d*t*)^2^) now follows a distribution P(*s*) = *s*^*−τ*^exp(−*s/s*_1_)(1 − exp(−*s/s*_2_))^44^ with *τ = *1.3, *s*_1_ = 2.68 and *s*_2_ = 15.64 where the high energy cut-off *s*_1_ dominates. Waiting time analysis shows the same sequence (Fig. 3d–f): the first two scenarios show power law distributed waiting times (normalized waiting time: waiting time/<waiting time>) with exponents 1.95 and 1.92. The third scenario yields an exponential distribution P(*s*) = 0.29 ∗ exp(−*s*/*s*_*o*_) with *s*_*o*_ normalized by the average waiting time. In the Supplementary Fig. S4, we show how the exponents are estimated by Maximum Likelihood method^44,45^ for stress drops and waiting times.

### Deformation characterization

The deformation mechanisms of the three β-Ti alloy pillars are analysed by (scanning) transmission electron microscopy ((S)TEM). We observe that deformation of Ti-32Mo pillar is mediated by dislocation slip (Supplementary Fig. S5), which is typical for BCC alloys, and shows large avalanche amplitudes in stress-strain curves^7,41^. Precipitates hinder such dislocation movements in the slip plane of ω_iso_-Ti-20Mo pillars (Supplementary Fig. S6). Further loading increases the stress concentration in front of ω_iso_ precipitate, and leads to dislocations cut through the precipitate^42,46^. The movement of dislocations is reduced and avalanche amplitudes are smaller than in the first scenario.

**Figure 4:**
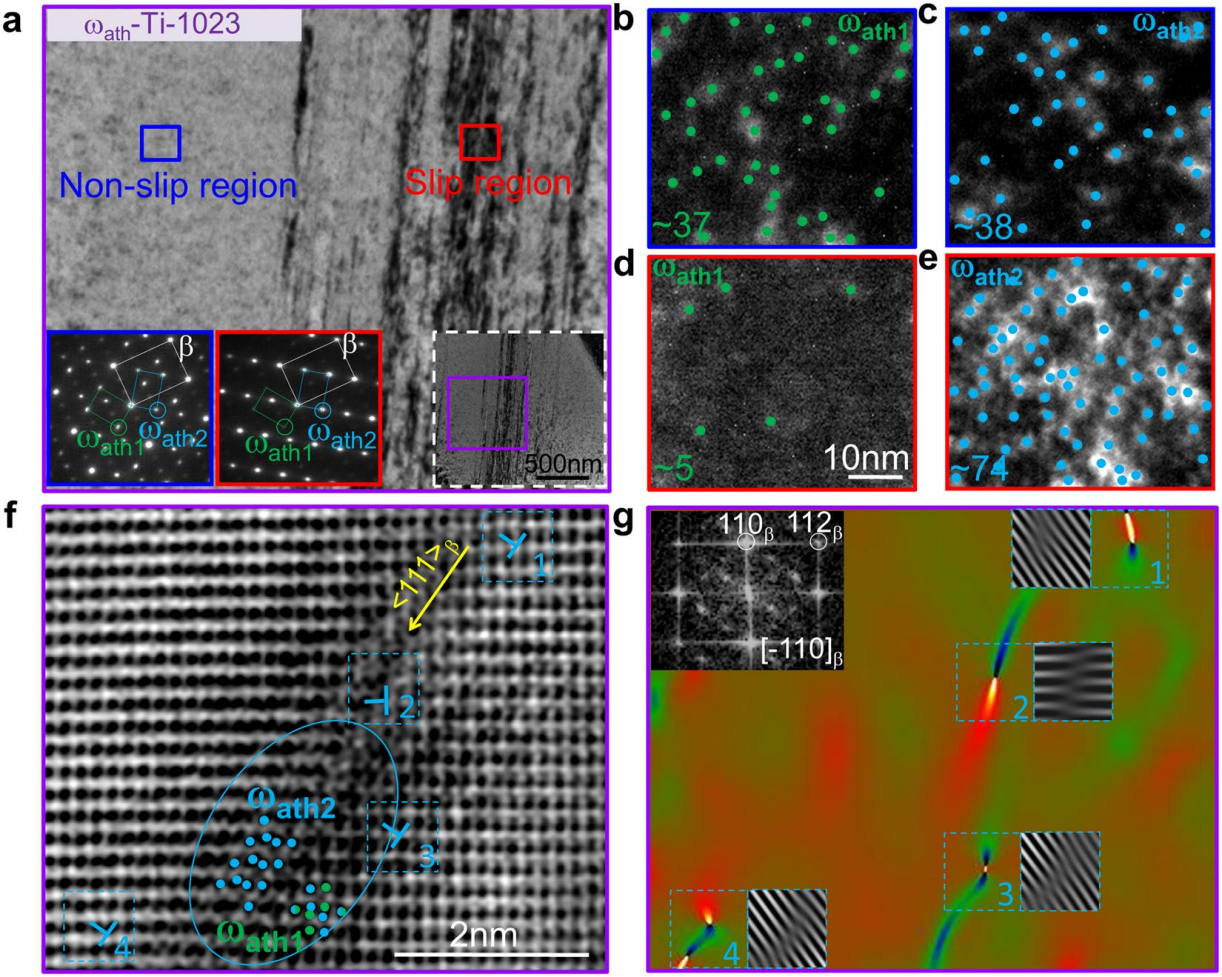
Deformation features of ω_ath_-Ti-1023 pillars with 5% compression strain. (**a**) Bright-field image of deformed pillar with many slip lines, which is the enlarged view of the purple square area of the lower-right inset containing the whole slip region. The blue and red rectangles display typical non-slip and slip regions. The lower-left insets correspond to selected area electron diffraction (SAED) patterns along the <011>_β_ zone axis showing two ω_ath_ variants reflections. The ω_ath1_ and ω_ath2_ are two crystallographic variants of the ω_ath_ phase. (**b**,**c**) Dark-field images using the ω_ath1_ and ω_ath2_ reflections corresponding to the blue rectangle, which shows that the number of ω_ath1_ and ω_ath2_ precipitates is almost the same (~38) in non-slip region. These white speckles are the ω_ath_ precipitates. White ω_ath1_ and ω_ath2_ precipitates are highlighted by green and blue dots, respectively. (**d**,**e**) Dark-field images corresponding to the red rectangle, which shows that in slip region the number of ω_ath1_ precipitates is near to zero and the number of ω_ath2_ (~74) is increased as double as the non-slip region. (**f**) STEM annular bright-field (ABF) lattice image shows dislocations pile-up in front of ω_ath_. They induce ω_ath_ variant rotation and produce new dislocations on the rear of the rotated ω_ath_ variant. Typical ω_ath1_ and ω_ath2_ crystal lattices are highlighted by six green and blue dots, respectively. The yellow arrow shows the slip direction. (**g**) Detailed characterization of the dislocations around ω_ath_ precipitate in (**f**) by Geometric phase analysis (GPA) choosing (110)_β_ and (112)_β_ to calculate strain. Strain regions are highlighted by rectangles. The inset in the top left shows the fast Fourier transform (FFT) results of (**f**). The inset of two inverse FFT images with (110)_β_ and (112)_β_ reflections confirm that the highlighted strain regions by GPA are regions with dislocation.

Rotations of ω_ath_ precipitates occur in ω_ath_-Ti-1023 pillars under applied stress. TEM images of a ω_ath_-Ti-1023 pillar with 5% compressive strain show many parallel slip planes (Fig. 4a). Figure 4a is the enlarged view of the purple square area of the lower-right inset. It is clearly seen that the slip region in Fig. 4a stems from the deformed area. Selected area electron diffraction (SAED) of the non-slip region (blue rectangle in Fig. 4a) show that the diffraction intensities of two equivalent ω_ath_ variants (ω_ath1_ and ω_ath2_) are almost identical. Dark-field TEM images using ω_ath1_ and ω_ath2_ reflections (Fig. 4b,c) further prove that the concentrations of ω_ath1_ and ω_ath2_ precipitates in the non-slip region are identical. The SAED patterns of the slip region show only a weak diffraction intensity of ω_ath1_, and a strong diffraction intensity of ω_ath2_ (red rectangle in Fig. 4a). The related dark-field TEM images using ω_ath1_ and ω_ath2_ reflections (Fig. 4d,e) show that ω_ath1_ precipitates almost disappeared, while the number of ω_ath2_ approximately doubled. The absence of the ω_ath1_ inside slip planes indicates a transformation from ω_ath1_ to ω_ath2_ under stress. STEM annular bright-field (ABF) lattice images of the slip region (Fig. 4f) also show that ω_ath1_ variant rotate to ω_ath2_ variant. The ω_ath_ phase is identified by the atomic structural feature, which is used in reference^40^. Typical ω_ath2_ crystal lattices are highlighted by six blue dots. There is no sharp boundary between β matrix and ω_ath_ phase as the structural similarity and coherent nature of the interface. The blue ellipses in Fig. 4f are approximate indications of the ω_ath2_ phase boundaries. Along the slip direction, ω_ath2_ is preferred, while ω_ath1_ is not preferred from Fig. 4d,e. However, we do find that some ω_ath1_ structures (represented by six green spots) still exist in the ω_ath2_ phase region. As the average ω_ath_ lattice parameter is 3.27 nm, the fact that ω_ath2_ and ω_ath1_ phase co-exist within 2 nm-region along the slip bands indicates some ω_ath1_ phase is not rotated and retains its previous orientation. The Burger’s vector orientation of most dislocations around ω_ath_ precipitate are consistent with the slip direction (shown in Fig. 4f,g), while the orientation of dislocation (numbered 2) in front of ω_ath_ precipitate derivate from the slip direction, where the dislocation is obstructed by the ω_ath_ precipitate along its pathway. The accumulation of dislocations in front of ω_ath_ precipitate will lead to an obvious lattice distortion and thus its Burger’s vector orientation is different from other dislocations. Therefore, the variant rotation is activated by stress concentration of dislocation pile-ups in front of ω_ath_ inclusions, and then facilitates the nucleation of new dislocations (indicated by blue dotted boxes in Fig. 4f). The propagation of dislocations is now blocked and restarts on the other side of the rotated ω_ath_ variant (or other sites where the stress concentration is high) with further loading, leading to many tiny slip lines, but no big slip bands.

To further verify the existence of dislocations around ω_ath_ precipitate, the ABF lattice image (Fig. 4f) was analyzed by Geometric Phase Analysis (GPA)^47,48^. GPA technique is described in the Supplementary Note 1. Figure 4g shows the GPA results choosing (110)_β_ and (112)_β_ to calculate strain. The butterfly-like strain field in the selected region (with blue dotted line in Fig. 4g) reveals the positions of dislocation core. The related insert inverse fast Fourier transform images further confirm these strain regions are close to dislocations.

## Discussion

### The precipitate effect on plasticity stability

**Figure 5:**
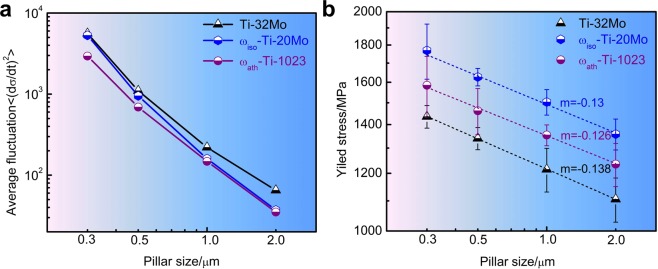
Comparison of average fluctuation with sample size (**a**) and size-dependent yield stress at 0.2% plastic strain for three β Ti-alloy pillars (**b**).

In this work, the jerk energy is defined as the square of stress drop rate, (d*σ*/d*t*)^2^. Figure 5a shows that the average jerk energy decrease from Ti-32Mo to ω_iso_-Ti-20Mo at the large pillar size, while the reduction of jerk energy becomes uncertain at small pillar sizes. However, ω_ath_ reduces the jerk energies and improves the plasticity stability in ω_ath_-Ti-1023 at all pillar sizes. The reason is that the ω_ath_ precipitates act as pinning and nucleation centers in the slip region as described above. The rotation of ω_ath_ precipitates strongly pins and blocks the avalanche movement of dislocations. Dislocations can now only glide over small distances. The rotation of ω_ath_ precipitates also leads to dislocation nucleation with high rates. Supplementary Fig. S7 shows dense slip traces occurs in ω_ath_-Ti-1023 pillar in comparison with Ti-32Mo and ω_iso_-Ti-20Mo pillars, which indicates large conservation of dislocations in ω_ath_-Ti-1023.

The rotation of ω_ath_ precipitates is changed in Ti-1023 sample to non-rotatable ω_iso_ precipitates (called ω_iso_-Ti-1023) through heat treatment (aging at 250 °C for 1 hour)^49,50^. The ω_iso_ precipitates have the similar mean diameter of 3.5 nm and mean spacing of 6.75 nm with ω_ath_ precipitates in Ti-1023 as show in Supplementary Fig. S8. Supplementary Fig. S9 shows obvious stress drops with the probability distribution function of both stress drop and waiting time in ω_iso_-Ti-1023 pillars now following the power law distribution (see Supplementary Fig. S10): P(*s*) **∼** *s*^−*τ*^ with *τ* ∼ 2 as in ω_iso_-Ti-20Mo pillars. Thus, the various mechanical behavior of the micropillars mainly depends on the states of the ω precipitates.

### The size effect of the yield stress

The size effects of yield stress *σ*_0.2_ (stress at 0.2% plastic strain) for three β Ti-alloy pillars are shown in Fig. 5b. We find that “smaller is stronger” is still valid in all the three cases. The *σ*_0.2_ follows a power-law dependence of pillar width D, *σ*_0.2_~*D*^*m*^, and the best fitted m values are close for all the three cases. The size effect is weaker in ω_ath_-Ti-1023 alloy due to the revolving mechanism of precipitates which facilitate more nucleation of mobile dislocations. Size-dependent strength of precipitated alloys has been investigated in duralumin micropillars^51^. It was found that the mean spacing of the precipitates, as an internal length scale, governs the mean free path of mobile dislocations in the micropillar during deformation, and then determines the strength. The interplay between precipitate strength and dislocation starvation also gives rise to a “weakest size” phenomenon. In the present case, both ω precipitate size and mean spacing are nanoscale in Ti-alloys (Supplementary Fig. S1), however. It takes a large number of precipitates to enhance the pillar strength even in small pillars. All yield stresses *σ*_0.2_ of pillars are larger than the yield stress of ω_iso_-Ti-20Mo bulk (Fig. S11, 966 MPa). This indicates that there is no “weakest size” for the three cases.

### The effect of deformation on the fluctuation statistics

Our experiments show that the deformation behavior is strongly correlated with the jerk statistics. For Ti-32Mo alloy without visible precipitates, dislocations can move freely after nucleation and travel to the free surface easily, forming slip bands. During the motion, dislocations can also trigger other dislocations to slip near the original slip plane^21^, which makes the plastic deformation to be mainly concentrated on a few of slip bands as seen in Fig. 2d. Thus, these correlated dislocation motions are self-organized with broad power-law-distributed statistics^3^, which is scale-free. For ω_iso_-Ti-20Mo, the precipitates can block the movements of dislocations. Further loading will lead to dislocations cut through the ω_iso_ precipitates, and dislocations also travel to the free surface, forming slip bands. Finally, dislocation motions share the same characteristics with Ti-32Mo. Therefore, the first two scenarios show little difference, including the statistics and the deformation morphology. But for ω_ath_-Ti-1023 pillar, there is no slip localization, and the slip lines are on average uniformly distributed throughout the sample. The dislocation motions are blocked by ω_ath_ precipitates and randomly restarts on the other side of the rotated ω_ath_ variant or other sites where the stress concentration is high with further loading. Plastic flow then proceeds mainly through small and uncorrelated dislocation motion, confined inside the transient ω_ath_ variants, which give rise to small and uncorrelated stress fluctuations that is scale dependent. Eventually, the statistics changes from power-law to exponential when ω_ath_ precipitates are contained in the pillar.

## Conclusion

In summary, we report that highly anisotropic ω_ath_ phase inclusions, under appropriate conditions, rotate - similar to a revolving door - influencing the progression of dislocations and hence the formation of avalanches. The surprising result of our investigations is the magnitude of the effect: rotors greatly reduce crackling noise and, even more perplexing, change their statistical fingerprint from power laws to exponentials.

## Methods

### Sample preparation

The virgin Ti-10V-2Fe-3Al alloy sample was solution-treated at 830 °C for 24 hours in vacuum, followed by water quenching to room temperature. The microstructure is a coarse β phase and a high density of athermal ω precipitates (designated as ω_ath_-Ti-1023). The virgin Ti-20Mo alloy sample was first solution-treated at 1000 °C for 1 hour and quenched in water. The sample was then aged at 350 °C for 1 hour in vacuum, followed by air cooling. The microstructure is a β phase with isothermal ω precipitates (designated as ω_iso_-Ti-20Mo). The specimen of Ti-32Mo alloy was homogenized at 900 °C for 0.5 hour in vacuum, and followed by air cooling. The corresponding microstructure shows β phase without visible precipitates. Details of the above microstructures are shown in the Supplementary Fig. S1.

The crystallographic orientations of Ti-32Mo, ω_iso_-Ti-20Mo and ω_ath_-Ti-1023 alloy specimens were measured using Oxford NordlysNano electron backscatter diffraction (EBSD) detector. Single-crystal pillars with a square cross-section were then fabricated from the [011]-oriented grains in bulk specimens, using a dual Focus Ion Beam(FIB) milling and scanning electron microscope (SEM) in a FEI Dual Beam Helios NanoLab 600 instrument. The width of pillars ranges from 300 nm to 2 μm with an aspect ratio of **∼**2.5.

### Testing and characterization

Quantitative compression test was carried out in a Hysitron Ti-950 with a 10 μm flat-tip diamond punch in displacement-controlled mode at a constant nominal strain rate of 5 × 10^−4^/s. In order to ensure the reliability of data, the thermal drift usually was controlled to below 0.05 nm/s in the compression tests. The cross-sectional area at top surface of the pillar and the initial height were used to calculate engineering stresses and strains. The TEM lamellas of micro-pillar were prepared using the FIB lift-out technique for microstructural observation. The orientation of lamellas remains similar for all samples. Microstructural observations were performed with a JEOL-2100F high-resolution TEM and a FEI TF30 aberration-corrected STEM.

### Statistic methods

Stress time series are shown in Supplementary Fig. S3a for a 0.3 μm Ti-32Mo micro-pillar. Jerk signals were identified by numerically differentiating the flow stress time series *σ*(*t*) (Fig. S3b). The average jerk amplitude (dσ/dt) at the unloading stage was used as a threshold to exclude the background noise of the instrument. The threshold is about 7.72 MPa/s for 300 nm pillar, as shown in the inset of Fig. S3b. The positive peaks of d*σ*/d*t* curve were filtered out as they correspond to the stress rises in the flow stress time series (Fig. S3c). An avalanche begins when the value of d*σ*/d*t* curve falls below the threshold (*t*_1_ in Fig. S3e) and ends when the derivative increases to above the threshold (*t*_2_ in Fig. S3e) and then restarts when the derivative falls below the threshold again (*t*_3_ in Fig. S3e). The waiting time is defined as the difference between *t*_2_ and *t*_3_, that is *t = t*_3_ − *t*_2_^14,19^. The jerk amplitude is defined as the height of peak in the squared derivative time series (d*σ*/d*t*)^2^ (Fig. S3d,f)^30,31^. The probability to find a certain jerk amplitude (or waiting time) is plotted as a function of amplitude (or waiting time) as Probability Distribution Function (PDF). We applied the Maximum Likelihood (ML) method to determine the power-law exponent^44,52^.

## Supplementary information

Rotatable precipitates change the scale-free to scale dependent statistics in compressed Ti nano-pillars

## Data Availability

The data that support the findings of this study are available from the corresponding authors upon reasonable request.
