# Supplementary material for: Rotatable precipitates change the scale-free to scale dependent statistics in compressed Ti nano-pillars
Source: Sci Rep. 2019 Mar 7;9:3778. doi: 10.1038/s41598-019-40526-5 (PMC6405840; doi:10.1038/s41598-019-40526-5)
Supplement: Supplementary file 1 — Rotatable precipitates change the scale-free to scale dependent statistics in compressed Ti nano-pillars [file 41598_2019_40526_MOESM1_ESM.pdf]

Supplementary material

Rotatable precipitates change the scale-free to scale dependent statistics in compressed Ti nano-pillars

Yan Pan[1], Haijun Wu[2], Xiaofei Wang[1], Qiaoyan Sun[1], Lin Xiao[1], Xiangdong Ding[1,*], Jun Sun[1] & Ekhard K. H. Salje[1,3,*]

[1]*State Key Laboratory for Mechanical Behavior of Materials, Xi'an Jiaotong University, Xi'an, 710049, China*

[2]*Department of Materials Science and Engineering, National University of Singapore 117575, Singapore*

[3]*Department of Earth Sciences, University of Cambridge, Cambridge CB2 3EQ, United Kingdom*

[*] Correspondence and requests for materials should be addressed to X. D. (email: dingxd@mail.xjtu.edu.cn) or E. S. (ekhard@esc.cam.ac.uk)

Supplementary 1: Microstructural analysis

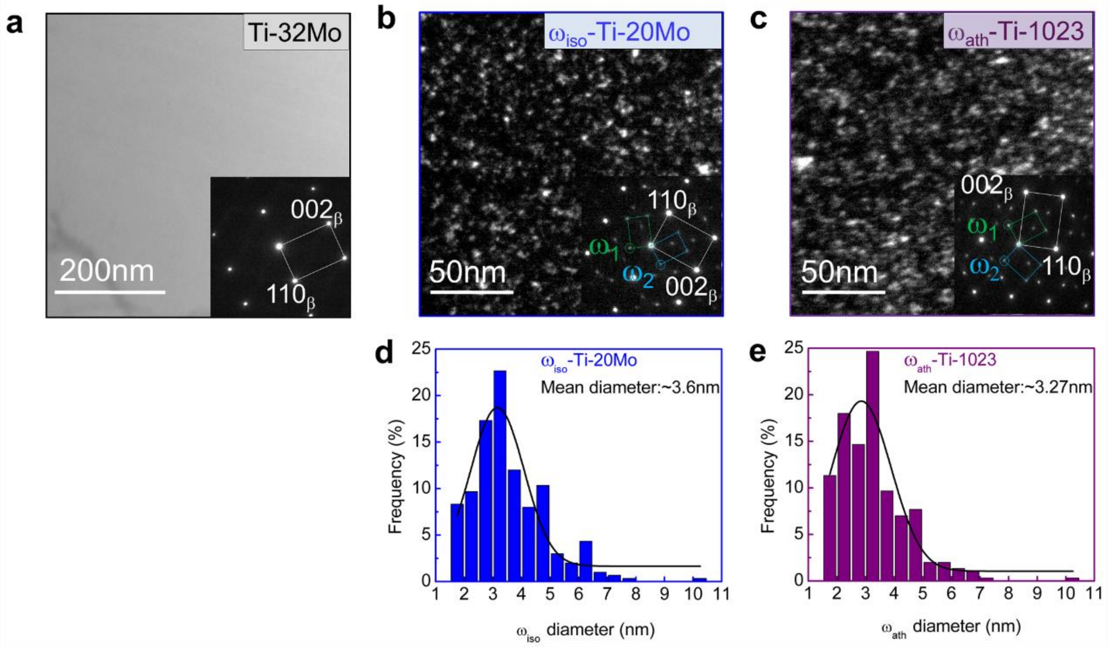

Figure S1. Microstructural characteristics of β-Ti alloys. (a-c) Bright field TEM image of β Ti-32Mo alloy without visible precipitates (a), dark field TEM image of β Ti-20Mo alloy with ω_{iso} precipitates (ω_{iso}-Ti-20Mo) (b), and dark field TEM image of β Ti-1023 alloy with ω_{ath} precipitates (ω_{ath}-Ti-1023) (c). Insets are the corresponding selected area electron diffraction (SAED) pattern. These ω precipitate reflections show two variants, as denoted by ω_1 and ω_2, the diffractions of ω_3 and ω_4 are not distinguished as they are overlapped with β diffraction. (d, e) The statistical results of ω precipitates size in ω_{iso}-Ti-20Mo alloy (b) and ω_{ath}-Ti-1023 alloy (c), respectively. The average diameters of ω_{iso} in Ti-20Mo and ω_{ath} in Ti-1023 are 3.6 and 3.27nm, respectively.

It is calculated precipitate size for ω_{iso} in Ti20Mo and ω_{ath} in Ti1023 alloy according to TEM image in Fig.S1 (b,c). However, we find there is no significant difference on the mean spacing and mean size of ω precipitate in ω_{iso} in Ti-20Mo and ω_{ath} in Ti-1023 alloy. The detail calculation is follows:

a) The precipitate size for ω_{iso} in Ti-20Mo are quantified as follows:

The mean size of ω_{iso} precipitates ($\bar{d}_{\omega}$) is ~3.6;

The mean spacing of ω_{iso} $l_{\omega} = \sqrt[3]{3/(4\pi\bar{N}_{\omega})}$ [1]; where the volumic numerical

density $\bar{N}_\omega = N_\omega / (\varsigma + \bar{d}_\omega)$ [2,3], ς is the TEM foil thickness (~50nm in our case), N_ω is the areal numerical density of all precipitates which can be determined by the approach of Yano [1] (N_ω~4.95×10^{-2} nm^{-2} in our case);

As a result, the calculated mean spacing of ω_{iso} in Ti-20Mo is l_ω= 6.37nm.

b) Using the equations, We find

the mean size of ω_{ath} precipitates in Ti1023 ($\bar{d}_\omega$) is ~3.27nm;

and the mean spacing of ω_{ath} precipitates in Ti1023 l_ω=7.9nm.

Supplementary 2: Mechanical behaviors of pillars

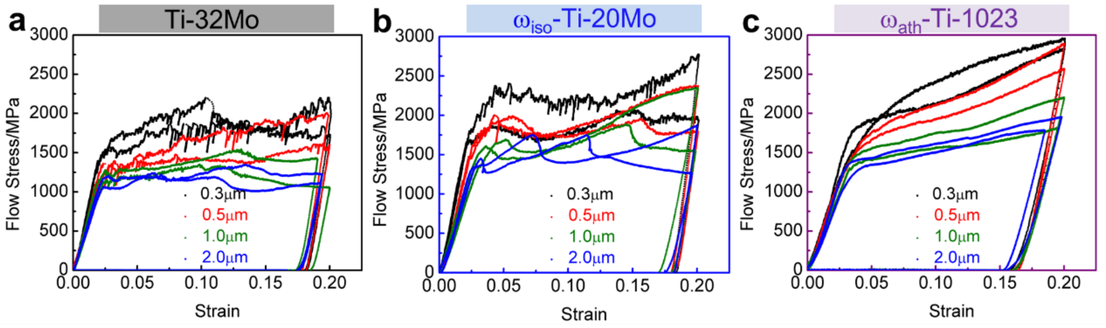

Figure S2. Representative stress–strain curves for three β-Ti alloys with pillar sizes ranged from 0.3-2.0 μm.

Supplementary 3: Statistics method of stress drop and waiting time

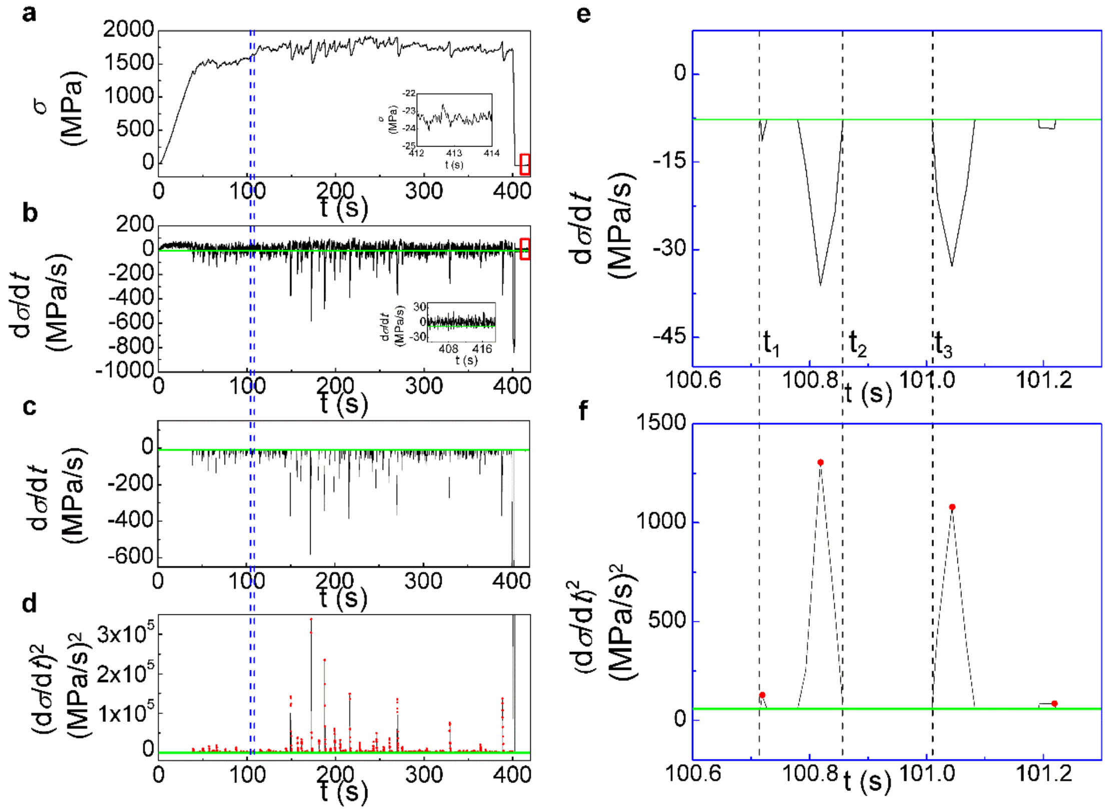

Figure S3. Data collection method. (a, b) Flow stress~time series of a Ti-32Mo nano-pillar with 300 nm diameter (a) and corresponding stress rate series (dσ/dt ~ t) (b). Inset is magnification of the partial unloading stage. The green line is the threshold =7.72 MPa/s. (c) The negative part of stress rate series (dσ/dt) before the unloading stage indicate the stress drop behavior. (d) Square of the negative part of the (dσ/dt) series, ((dσ/dt)^2). (e) Magnification of the negative part of (dσ/dt) series between the two blue dash lines in (c). These t_1, t_2 and t_3 are three successive times, which define the avalanche start time, end time and restart time, respectively. The time interval between t_2 and t_3 is the waiting time between two stress drops. (f) Magnification of the squared stress rate series ((dσ/dt)^2) between the two blue dash lines in (d). The red dots are the identified peaks, which represent the amplitudes of jerks.

In this work, we checked the fluctuation of stress drop (dσ/dt) at the unloading stage in order to get the background noise of the instrument. We noticed in Figure. S3a that there do contain some vibrations as shown in the inset of Fig. S3a. The average jerky amplitude (dσ/dt) in the unloading region is about 7.72 MPa/s for 300

nm pillar, as shown in the inset of Fig. S3b. We therefore use the 7.72 MPa/s as the threshold to exclude the background noise of the instrument.

Supplementary 4: Exponents estimated by Maximum Likelihood method for stress drops and waiting times.

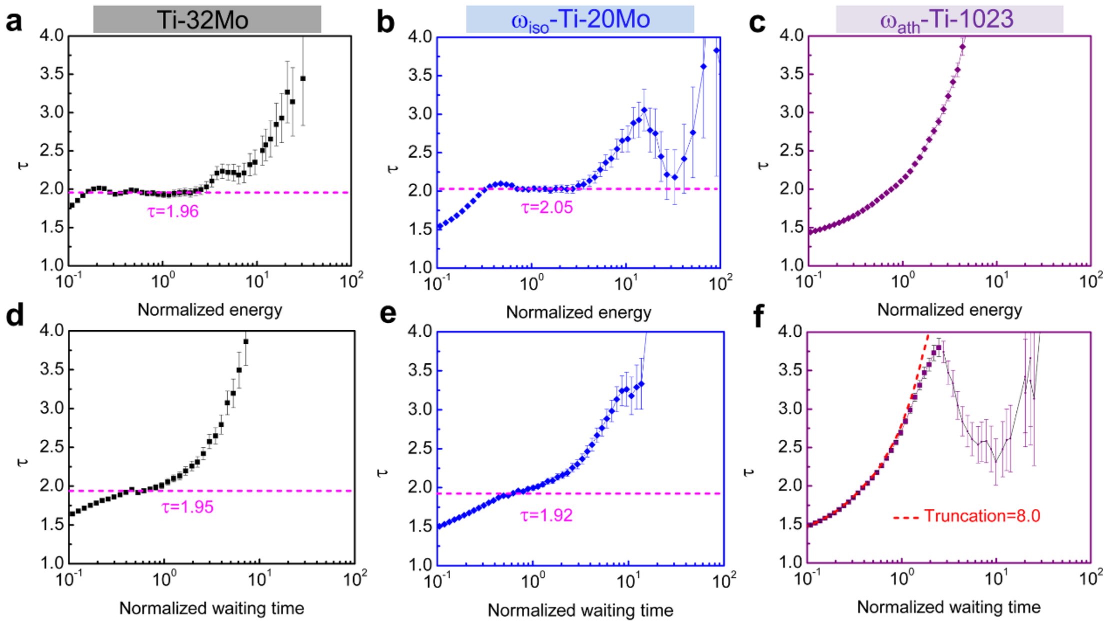

Figure S4. The Maximum Likelihood (ML) fitting exponent for stress drops and waiting times for the three scenarios in Fig.3. (a-c) ML-fitting exponents for stress drops for Ti-32Mo, ω_{iso}-Ti-20Mo, and ω_{ath}-Ti-1023 pillars, respectively. (d-f) ML-fitting exponents for waiting times for Ti-32Mo, ω_{iso}-Ti-20Mo, and ω_{ath}-Ti-1023 pillars, respectively. This analysis leads to a plateau that defines exponents for power-law distribution (a, b, d, e). There is no plateau for the Poisson distributions (e, f).

Fig.S4a,b show that the stress drops of Ti-32Mo and ω_{iso}-Ti-20Mo are power-law distributed with a plateau of about one decade, and the exponent τ~2 . However, for ω_{ath}-Ti-1023(Fig.S4c), the estimated τ rises continuously without a plateau.

The Maximum Likelihood method gives a similar result for waiting time

distributions (Fig.S4d-f). Fig.S4 (d,e) show that the waiting time of Ti-32Mo and ω_{iso}-Ti-20Mo are power-law distributed with the exponent τ~2. For ω_{ath}-Ti-1023 (shown in Fig.S4f), the exponent decreased when the normalized waiting time is between 2 and 10, due to the large tail in the PDF (Fig.3f in the main text). If we filter out the data larger than 8.0 in Fig.3f, the estimated exponent goes up continuously (Fig.S4f), which is similar to the exponent distribution of Fig.S4c for ω_{ath}-Ti-1023.

Supplementary 5: Microscopic observation.

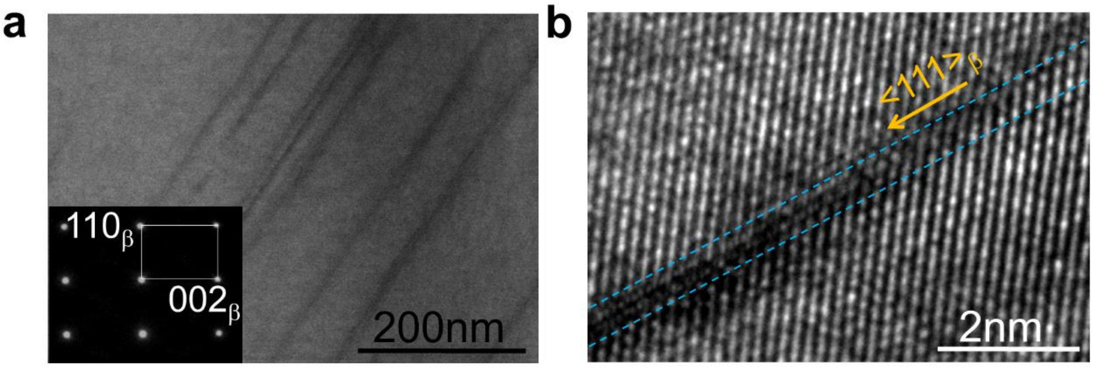

Figure S5. TEM morphologies showing the deformation features for Ti-32Mo pillar. (a) Bright-field image showing slip traces in the deformed Ti-32Mo pillar. Inset of SAED pattern along <011>$_\beta$ zone axis showing no ω reflections. (b) High-resolution TEM (HRTEM) show the traces of dislocation movement inside slip bands, leaving a large number of lattice misfits. The yellow arrow shows the <111>$_\beta$ slip direction.

Fig. S5 shows the TEM morphologies in the deformed Ti-32Mo pillars. Fig.S5a shows that, for bcc Ti-32Mo without visible precipitates, several parallel dislocation slip bands were formed in the deformed pillar. We further distinguish dislocation movements traces inside slip bands from HRTEM images (Fig.S5b), and found that a large number of lattice misfits remains in the deformed Ti-32Mo pillar (indicated by blue dashed lines).

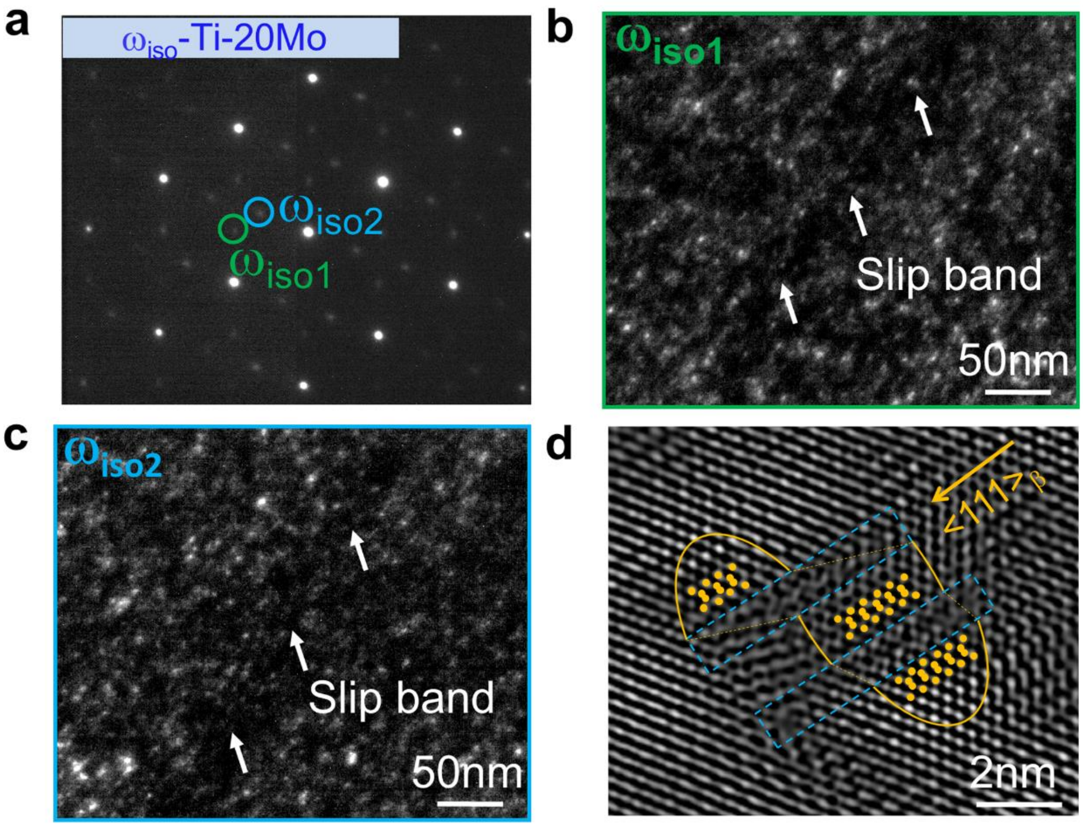

Figure S6. TEM morphologies showing the deformation features for ω_{iso}-Ti-20Mo pillar. (a) SAED pattern along <011> $_\beta$ zone axis showing two ω_{iso} reflections in the deformed ω_{iso}-Ti-20Mo pillar. (b, c) Two dark-field images using the ω_{iso1} and ω_{iso2} reflections in (a). The numbers of ω_{iso1} and ω_{iso2} were decreases in slip bands pointed by white arrows. (d) Fourier-filtered HRTEM with all main reflections shows that ω_{iso} precipitate is cut by dislocations and extra semi-coherent interfaces (in blue dash box) are produced in ω_{iso} precipitate along the slip plane. Typical ω_{iso} lattices are highlighted by yellow spots. The yellow arrow shows the <111>$_\beta$ slip direction.

Fig. S6 shows the TEM morphologies in the deformed ω_{iso}-Ti-20Mo pillars. For ω_{iso}-Ti-20Mo pillar, these ω_{iso} precipitate reflections reveal two variants, as denoted by ω_{iso1} and ω_{iso2} in Fig.S6a.The dark-field TEM images of ω_{iso1} and ω_{iso2} reflections are show in Fig.S6b,c, respectively. The numbers of ω_{iso1} and ω_{iso2} decrease sharply along slip bands, forming a black slip band (as indicated by white arrows). Fourier-filtered HRTEM with all main reflections shows that ω_{iso} precipitate is cut by dislocations and extra semi-coherent interfaces are produced in the ω_{iso} precipitate

along the slip plane (Fig.S6d)

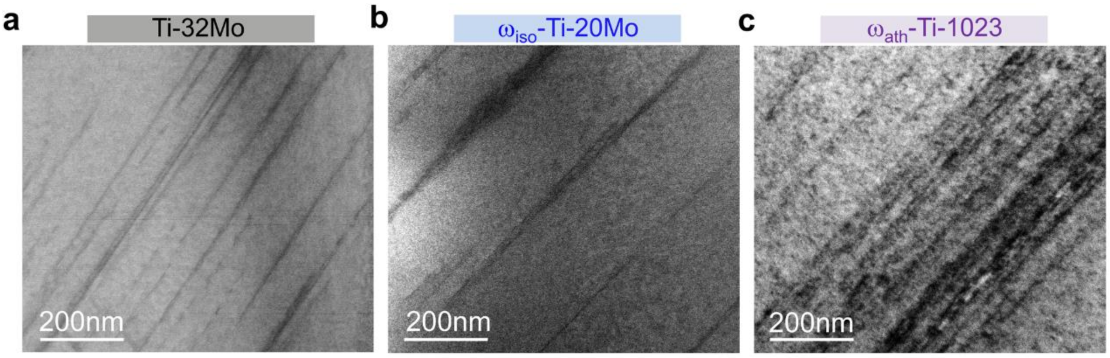

Figure S7. Comparison of TEM morphologies taken from the three deformed pillars. Bright field TEM images of Ti-32Mo (a), ω_{iso}-Ti-20Mo (b), and ω_{ath}-Ti-1023 (c).

Supplementary 6. Compressive behavior and statistics of stress drop and waiting time for ω_{iso}-Ti-1023 alloy.

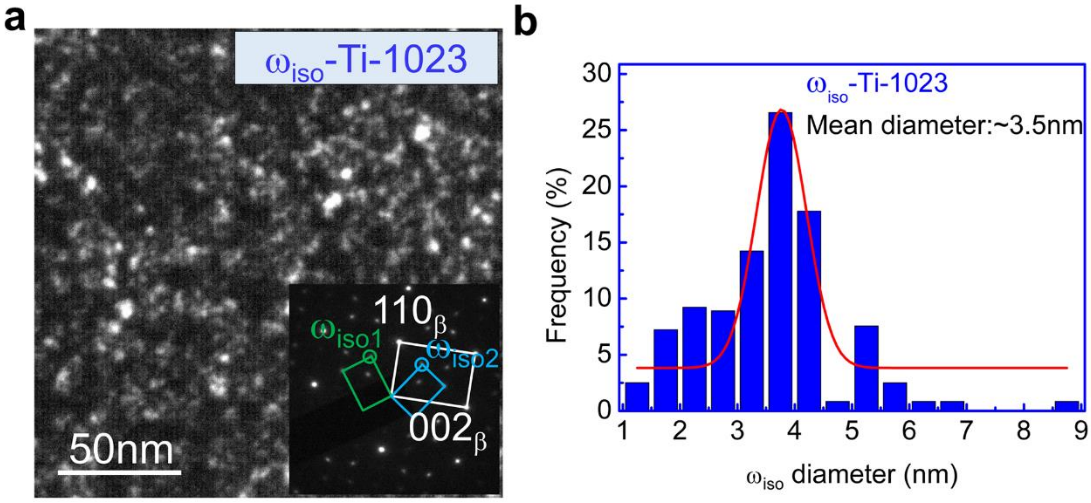

Figure S8. Microstructural characteristics of ω_{iso}-Ti-1023 alloy. (a) Dark field TEM image of ω_{iso} precipitates and corresponding SAED pattern. (b) The statistical results of ω_{iso} precipitates size.

From Fig.S8, we find the mean size of ω_{iso} ($\bar{d}_\omega$) =3.5nm, and mean spacing of ω_{iso} (l_ω) =6.75nm; while the mean size of ω_{ath} ($\bar{d}_\omega$)=3.27nm, and mean distance of ω_{ath} (l_ω)=7.9nm from Fig S1(c,e). In present case, the ω_{iso} keep the similar mean size and

spacing with the ω_{ath}. We did additional experiments on the size effect of ω_{iso}-Ti-1023 pillars, and we find that the yield stress (at 0.2% plastic strain) still follow the trend of “smaller is stronger” (Fig.S9b). Similar size effect has been reported in ω_{iso}-Ti-1023 cylindrical pillars [1].

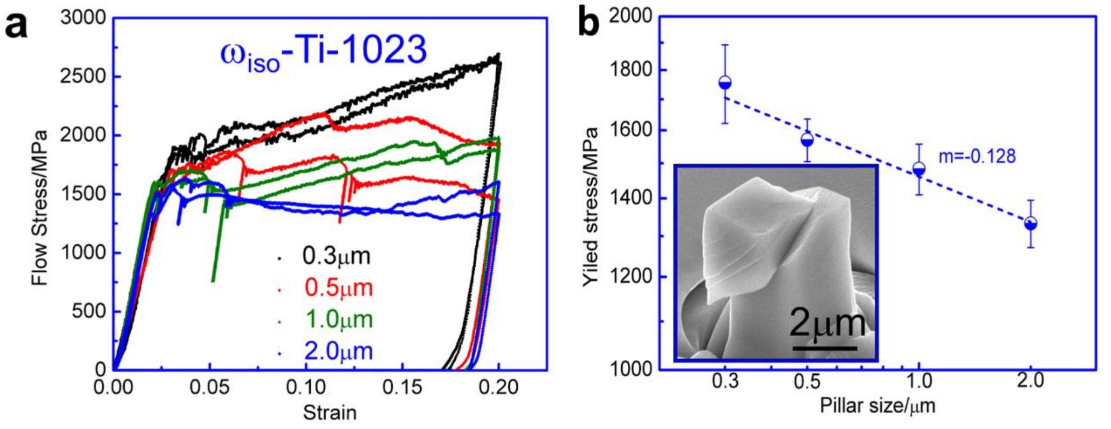

Figure S9. Compressive behavior for ω_{iso}-Ti-1023 pillars. (a) Representative stress–strain curves with pillar sizes ranged from 0.3-2.0μm. (b) Size-dependent yield stress at 0.2% plastic strain. Inset is the deformation characteristic of 2μm pillar.

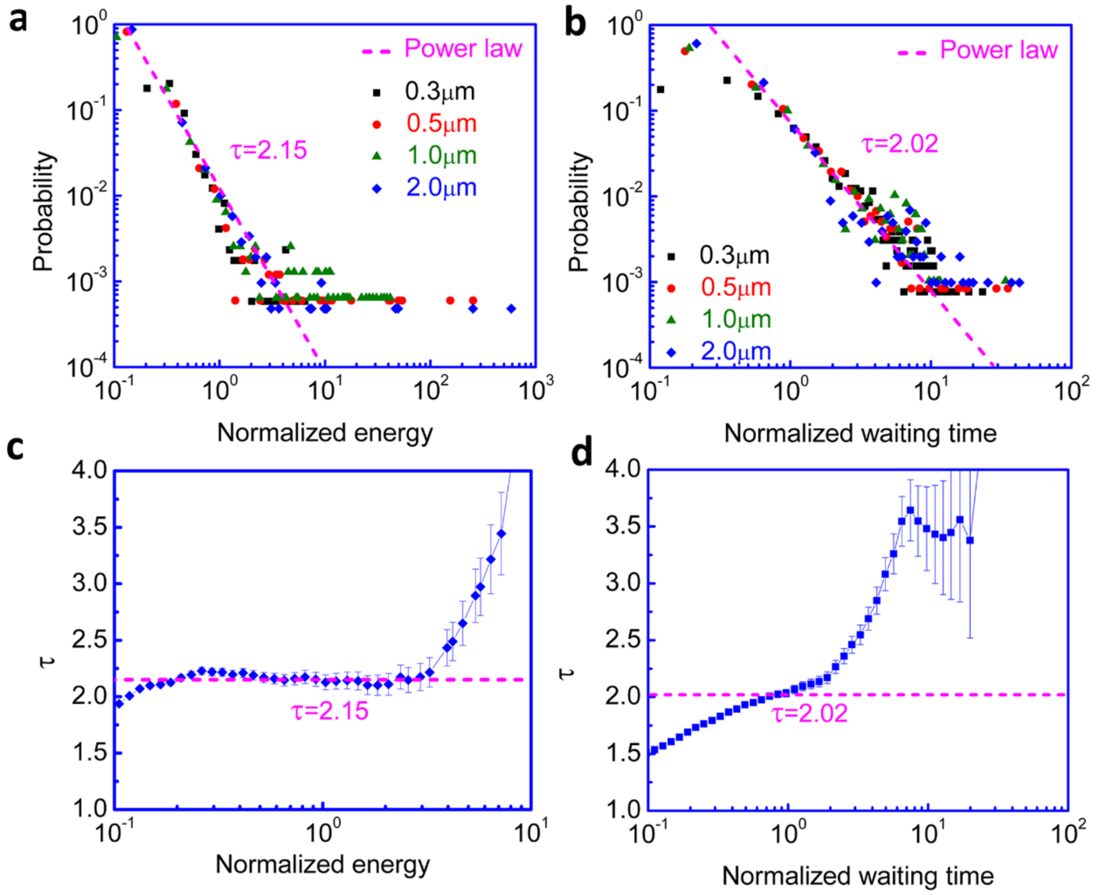

Figure S10. Statistics of stress drop and waiting time for ω_{iso}-Ti-1023 alloy. (a,b) Probability distribution functions for stress drops and waiting times for the pillars in Fig.S9a. (c,d) ML-fitting

exponents for stress drops and waiting times, showing the distributions of stress drop and waiting time follow the power law distribution with the exponent τ ~2.

Supplementary 7: Mechanical behaviors of bulk ω_{iso}-Ti-20Mo alloy

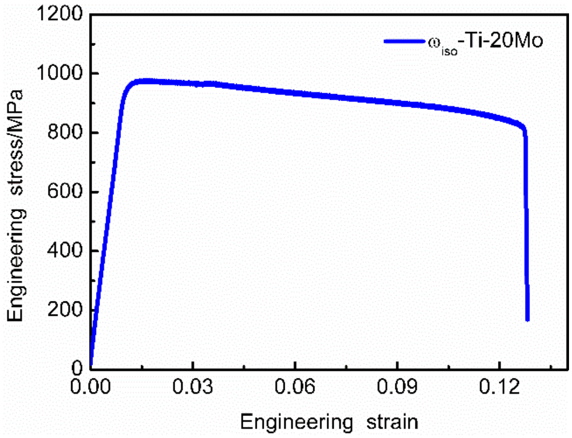

Figure S11. Engineering tensile stress-strain curve of bulk ω_{iso}-Ti-20Mo alloy, yield stress at 0.2% plastic strain is 966 MPa.

Supplementary Note1. Geometric phase analysis (GPA)

Geometric phase analysis (GPA)[4,5] is a method for measuring and mapping displacement fields and strain fields from high-resolution (scanning) transmission electron microscope (HRTEM/STEM) images, which is a semi-quantitative lattice image-processing approach for revealing spatially distributed strain fields. This technique allows map of the local distortion to be measured from the HRTEM/STEM images. The GPA is based on combining real-space and reciprocal-space information. Local strain components can be found by analysing the derivative of the displacement field. Displacements are measured by calculating the "local" Fourier components of the lattice fringes in an image.

References

[1] Yano, T. et al. Transmission electron microscopy studies on nanometer-sized ω phase produced in Gum Metal, *Scr. Mater.* **63**, 536–539 (2010).

[2] Cahn, J., Nutting J., Transmission quantitative metallography, *Trans. Metall. Soc. AIME* **215**, 526-528 (1959).

[3] Chen, W. et al. Strong deformation anisotropies of ω-precipitates and strengthening mechanisms in Ti-10V-2Fe-3Al alloy micropillars: precipitates shearing vs precipitates disordering, *Acta Mater*. **117**, 68-80 (2016).

[4] Hÿtch, M. J., Snoeck, E. & Kilaas, R. Quantitative measurement of displacement and strain fields from HREM micrographs, *Ultramicroscopy* **74**, 131-146 (1998).

[5] Xin, J. et al. Mg vacancy and dislocation strains as strong phonon scatterers in $Mg_2Si_{1-x}Sb_x$ thermoelectric materials, *Nano Energy* **34**, 428-436 (2017).
